# MicroRNAs Regulated by the LPS/TLR2 Immune Axis as Bona Fide Biomarkers for Diagnosis of Acute Leptospirosis

**DOI:** 10.1128/mSphere.00409-20

**Published:** 2020-07-15

**Authors:** Charles Solomon Akino Mercy, Natarajaseenivasan Suriya Muthukumaran, Prema Velusamy, Palanisamy Bothammal, Krishnamoorthi Sumaiya, Perumal Saranya, Dianne Langford, Santhanam Shanmughapriya, Kalimuthusamy Natarajaseenivasan

**Affiliations:** a Medical Microbiology Laboratory, Department of Microbiology, Centre for Excellence in Life Sciences, Bharathidasan University, Tiruchirappalli, Tamil Nadu, India; b Department of Biotechnology, School of Biotechnology and Genetic Engineering, Bharathidasan University, Tiruchirappalli, Tamil Nadu, India; c Heart and Vascular Institute, Pennsylvania State University, College of Medicine, Hershey, Pennsylvania, USA; d Department of Medicine, Pennsylvania State University, College of Medicine, Hershey, Pennsylvania, USA; e Department of Cellular and Molecular Physiology, Pennsylvania State University, College of Medicine, Hershey, Pennsylvania, USA; f Department of Neuroscience, Lewis Katz School of Medicine, Temple University, Philadelphia, Pennsylvania, USA; University of Kentucky

**Keywords:** leptospirosis, LPS, microRNA, TLR2, biomarkers, PPI network

## Abstract

Leptospirosis remains a significant human health issue due to its systemic complications. Therefore, biomarkers that are more effective are urgently needed for the early diagnosis of leptospirosis. MicroRNAs (miRNAs) are evolutionarily conserved regulatory RNAs that have shown the potential to be used as biomarkers for diagnosis, prognosis, and therapy of infectious diseases. In this study, we performed an unbiased screen using the miRNome miRNA array to identify circulating miRNAs with the potential to serve as authentic biomarkers for early diagnosis of leptospirosis. Because leptospiral lipopolysaccharide (LPS) is the predominant leptospiral antigen and plays a vital role in immunological and biological activities, we used LPS treated and untreated *in vitro* (THP1 cells) and *in vivo* (BALB/c mice) surrogate models to identify the LPS-specific miRNAs. Differential expression analysis revealed 18 miRNAs to be associated strongly with LPS stimulation in THP1 cells. Of these, three (miR-let-7b-5p, miR-144-3p, and miR-21-5p) were observed to be present at increased levels *in vivo*. The identified miRNAs were validated for their biomarker potential using serum samples from leptospirosis-negative patients and patients with confirmed cases of leptospirosis. Identified miRNAs were able to discriminate the acute leptospiral infection from other febrile diseases with a test sensitivity and specificity of 93.2% and 88.19%, respectively. Gene functional enrichment and protein-protein interaction (PPI) network analysis revealed that the identified miRNAs play important roles in disease signal transduction, signaling by interleukins, the stress-activated protein kinase signaling cascade, the mitogen-activated protein kinase (MAPK) signaling pathway, and the cellular response to a transforming growth factor β (TGF-β) stimulus with a notable interconnection between these biological processes.

**IMPORTANCE** Here, we used miRNAs that are differentially regulated by the LPS/TLR2 immune axis to devise a miRNA-based diagnosis for leptospirosis. The study established the role of the circulating stable miRNAs (miR-21-5p, miR-144-3p, and miR-let-7b-5p) as an early diagnostic marker for leptospirosis. These miRNAs can be used to diagnose acute leptospirosis and also to differentiate leptospiral infection from other bacterial and spirochetal infections, as proved by the use of human clinical samples. Thus, our findings indicate that miRNAs can play a crucial role in the diagnosis of infectious diseases, like leptospirosis, that are generally misdiagnosed.

## Abstract (precis)

Here, we used miRNAs that are differentially regulated by the LPS/TLR2 immune axis to devise a miRNA-based diagnosis for leptospirosis. The study established the role of the circulating stable miRNAs (miR-21-5p, miR-144-3p, and miR-let-7b-5p) as an early diagnostic marker for leptospirosis. These miRNAs can be used to diagnose acute leptospirosis and also to differentiate leptospiral infection from other bacterial and spirochetal infections, as proved by the use of human clinical samples. Thus, our findings indicate that miRNAs can play a crucial role in the diagnosis of infectious diseases, like leptospirosis, that are generally misdiagnosed.

## INTRODUCTION

Leptospirosis is a zoonotic disease caused by pathogenic serovars of spirochetal bacteria belonging to the genus *Leptospira* (1). Although leptospirosis has long been recognized as an important endemic disease in tropical countries, it is now becoming a more common problem in highly populated urban centers (2). The Leptospirosis Burden Epidemiology Reference Group (LERG) report included a systematic literature review that estimated the overall global annual incidence of endemic and epidemic human leptospirosis at 5 and 14 cases per 100,000 population, respectively (57). Leptospirosis ranges in severity from a mild flu-like illness to hepatorenal impairment, multiorgan failure, and septic shock with pulmonary hemorrhages. Clinically, it is typified by diverse symptoms that include fever, myalgia, headache, severe muscle pain, ocular disorders, meningitis, jaundice, renal failure, and pulmonary hemorrhage, and it is often confounded with other entities such as influenza and dengue fever. In this regard, early and definitive diagnosis of leptospirosis is critical for preventing its severe clinical complications.

We and others have determined that immunity against *Leptospira* depends on the production of circulating antibodies directed against serovar-specific lipopolysaccharide (LPS) (3, 4). *Leptospira* produces an atypical LPS that differs from LPS of Gram-negative organisms in several biochemical, physical, and biological properties (5). LPS is known to activate macrophages by a mechanism that involves the serum protein LPS-binding protein and a plasma membrane receptor complex comprising at least CD14 and one or more members of the Toll-like receptors (TLRs) (6–8). Among the 10 TLRs present in human, a large body of evidence supports TLR4 as the predominant receptor mediating LPS activation (9, 10). In contrast, it was shown recently that leptospiral LPS activates human cells through CD14 and TLR2 recognition (11, 12), whereas both TLR2 and TLR4 contribute to the activation of murine cells (11, 12). The recognition of LPS by TLRs results in subsequent recruitment of the intracellular adaptor protein MyD88 through homotypic Toll–interleukin 1 receptor (IL-1R) domains, leading to intracellular signaling through NF-κB and mitogen-activated protein kinase (MAPK) activation. The ultimate result of this LPS recognition by TLRs is a proinflammatory cytokine and chemokine response. However, when high levels of leptospiremia occur during infection, innate immune mechanisms eventually trigger tissue-based and systemic responses to infection that lead to severe outcomes, such as a sepsis-like syndrome or organ failure (13–15). Therefore, it seems probable that mechanisms exist to regulate the host immune response. Although several regulators have been proposed, microRNAs (miRNAs) are recognized as important regulators of immune response (16–18) and as fine tuners of TLRs (19–21).

miRNAs are 22-nucleotide-long small noncoding regulatory RNA molecules that regulate gene expression posttranscriptionally through complementary base pairing with thousands of messenger RNAs and play important roles in regulating diverse physiological, developmental, and pathophysiological processes (22). Alterations of miRNA expression profiles have been observed in various diseases, including cancer and cardiovascular, neurological, inflammatory, and autoimmune diseases. A growing body of evidence supports the key role of miRNA in the activation of both innate and adaptive immune response (23). Studies have shown differential expression of miRNA following LPS stimulation in various types of immune cells (23, 24). Several early and late LPS-responsive miRNAs have been reported (23, 24). Thus, identification of an miRNA repertoire responsive to TLR2-mediated LPS signaling will be a valid biomarker for the diagnosis of human leptospirosis. Identification of dysregulated miRNAs in infectious diseases is emerging to help devise novel diagnostics, prevention measures, and therapy (25). Additionally, extracellular miRNAs were identified to be circulating in the blood, thus raising the possibility of finding a connection between specific miRNA levels in serum and various disease states (26–28). Also, biochemical analyses indicate that miRNAs are resistant to RNase activity, extreme pH, extreme temperatures, extended storage, and large numbers of freeze-thaw cycles (26, 29, 30), making their isolation and analysis straightforward. Compared to protein-based biomarkers, miRNAs are homogenous and can be easily detected by qPCR, while low abundance and posttranslational modifications of protein markers affect the accuracy of the diagnosis (31). In this regard, the use of miRNAs that regulate LPS-TLR immune axis as novel biomarkers represents a new approach for early diagnosis of leptospirosis.

To that end, in the present study we used *in vitro* and *in vivo* models to identify the miRNAs that were differentially expressed during leptospiral LPS stimulation through the TLR2 axis. The identified miRNAs were validated for its efficacy to diagnose early leptospirosis using clinical samples. Additionally, we also predicted (i) the target genes, (ii) the potential functions of the differentially expressed miRNAs by gene ontology (GO) enrichment analysis, and (iii) a protein-protein interaction (PPI) network to identify the hub genes. The present study is the first to identify miRNAs specific for leptospiral LPS stimulation and provide a novel perspective on the involvement of these miRNAs in the pathological mechanism of leptospirosis.

## RESULTS

### Leptospiral LPS stimulates cell death and cytokine production.

**FIG 1:**
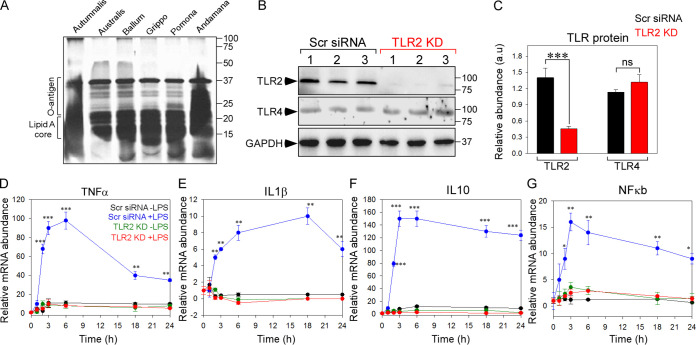
Extracted leptospiral LPS stimulates cytokine production in THP1 monocytes. (A) Representative SDS-gel image stained with silver stain. The electrophoretic mobility patterns of leptospiral LPS indicate apparent molecular masses between 14 and 37 kDa. (B) Representative Western blot analysis of the protein extracted from control (Scr siRNA) and TLR2 knockdown (KD) THP1 cells probed for antibodies specific for TLR2, TLR4, and GAPDH. The blot shows decreased expression of TLR2 and no changes in TLR4. (C) Quantification of the protein abundance of TLR2 and TLR4 from panel B by densitometric analysis, normalized to GAPDH. (D to G) Cytokine mRNA levels were measured in Scr siRNA and TLR2 KD cells treated with (blue and red) or without (black and green) LPS. Data are means ± SEM. ***, *P* < 0.001; **, *P* < 0.005; *, *P* < 0.01.

We first extracted and purified LPS from pathogenic (Leptospira interrogans serovar Australis strain AHF651, L. interrogans serovar Autumnalis strain N2, *L. kirschneri* serovar Grippotyphosa strain D22, L. interrogans serovar Pomona strain H3, and Leptospira borgpetersenii serovar Ballum strain BDU51) and nonpathogenic leptospiral strains (Leptospira biflexa serovar Andamana strain CH11). The concentration of the purified leptospiral LPS was between 575 and 625 μg/ml, and the LPS was free from protein, DNA, and RNA contaminations. The electrophoretic mobility patterns of leptospiral LPS evidenced apparent molecular masses between 14 and 37 kDa (Fig. 1A). Because leptospiral LPS is recognized by TLR2 in human cells due to differential recognition of the atypical lipid A moiety (11, 12), we transiently knocked down TLR2 in THP1 cells. Transient knockdown significantly decreased the levels of TLR2 in THP1 cells with no change in the TLR4 levels (Fig. 1B and C). Then, we verified the biological activity of leptospiral LPS using control (scrambled [Scr] small interfering RNA [siRNA]) and TLR2 knockdown cells. First, we measured the cytotoxicity of leptospiral LPS by MTT (3-(4,5-dimethyl-2-thiazolyl)-2,5-diphenyl-2H-tetrazolium bromide) assay. Increased cytotoxicity was observed in THP1 cells stimulated with pathogenic LPS compared to unstimulated or nonpathogenic LPS stimulation (Fig. S1A to 1F). Additionally, knocking down TLR2 in THP1 cells protected them against LPS-mediated cell death. Because we observed that LPS extracted from L. interrogans serovar Autumnalis strain N2 caused increased cytotoxicity compared to LPS from other pathogenic serovars, LPS from Autumnalis was used for further analysis. We then measured the mRNA levels of the proinflammatory mediators in control and TLR2 knockdown THP1 cells stimulated with Autumnalis LPS for various time points. In control THP1 cells, mRNA levels of tumor necrosis factor alpha (TNF-α), NF-κB, IL-1β, and IL-10 increased within 3 h of LPS stimulation. On the other hand, TLR2 knockdown significantly inhibited the elevated cytokine mRNA levels (Fig. 1D to G). This confirmed that extracted leptospiral LPS activated human cells through TLR2 recognition. Further, to better validate the reactivity of the extracted LPS *in vivo*, cytokines were measured by extracting mRNA from the whole blood of BALB/c mice at different time intervals (0, 4, 7, 14, and 21 days) after intraperitoneal injection of leptospiral LPS in phosphate-buffered saline (PBS). Consistent with the increase in cytokine mRNA levels in THP1 cells, at day 3 LPS postinjection, there was an increase in mRNA expression of proinflammatory mediators, further validating the potency of the extracted LPS (Fig. S2A to D).

10.1128/mSphere.00409-20.1 FIG S1 Extracted leptospiral LPS stimulates cell death in THP1 monocytes. Control (Scr siRNA) and TLR2 KD THP-1 cells were treated with LPS, and an MTT assay was performed. Scr siRNA THP1 cells treated with LPS showed increased cell death, whereas cell death was preserved in TLR2 KD cells. The percent cell survival was compared calculated using non-LPS-treated Scr siRNA and TLR2 KD THP1 cells. Download FIG S1, PDF file, 0.1 MB. Copyright © 2020 Akino Mercy et al. 2020 Akino Mercy et al. This content is distributed under the terms of the Creative Commons Attribution 4.0 International license.

10.1128/mSphere.00409-20.2 FIG S2 LPS stimulation upregulates cytokine production *in vivo*. (A to D) BALB/c mice were injected intraperitoneally (i.p.) with either PBS (black traces) or leptospiral LPS (red traces) reconstituted in PBS. After different intervals (0, 4, 7, 14, and 21 days), the cytokine profile was measured by qPCR analysis. The fold change in each target cytokine mRNA was normalized to the mouse β-actin housekeeping gene. Fold change in mRNAs of TNF-α (A), IL-1β (B), Il10 (C), and NF-κB (D) was measured. Data are means ± SEM. ***, *P* < 0.001; *, *P* < 0.05. Download FIG S2, PDF file, 0.1 MB. Copyright © 2020 Akino Mercy et al. 2020 Akino Mercy et al. This content is distributed under the terms of the Creative Commons Attribution 4.0 International license.

### miRNome miScript microarray analysis of miRNAs.

**FIG 2:**
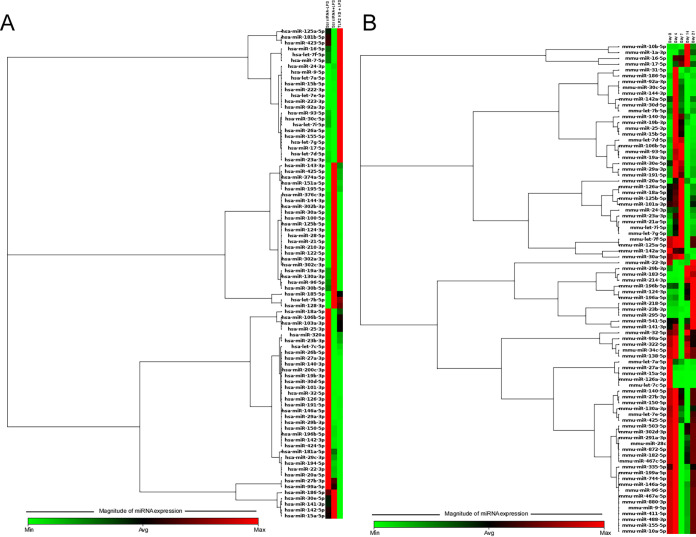
*In vitro* and *in vivo* miRNome miScript microarray analysis of miRNAs upregulated upon LPS treatment. (A) Hierarchical clustering of the differentially expressed miRNAs in control and TLR-2 KD THP1 cells after LPS stimulation. (B) Hierarchical clustering of the differentially expressed miRNAs in control and LPS-injected mice at different time intervals. Red represents miRNAs with an average fold change of 3, and green represents miRNAs with an average fold change of −3, relative to controls.

After confirming the ability of leptospiral LPS to prime immune responses through TLR2-dependent signaling, we next asked whether any of the positive or negative regulators of the TLR-signaling cascade could be used as biomarkers for the diagnosis of leptospirosis. Such a TLR regulation is primarily known to be achieved by the activation or repression of a large array of genes and microRNAs (miRNAs). Primarily, we used a human miRNome profile panel to identify LPS-responsive miRNAs that are differentially expressed in Scr siRNA and TLR2 knockdown THP1 cells. Analysis of the miRNA profile of the extracellular media of control and TLR2 knockdown cells with or without LPS treatment showed ∼18 miRNAs to be upregulated (>10-fold) in THP1 cells treated with LPS compared to untreated controls (Table S1 and Fig. 2A), whereas knocking down TLR2 normalized the upregulated miRNA levels, indicating that these miRNAs are specific to the TLR2-LPS immune axis and thus can serve as biomarkers (Table S1 and Fig. 2A).

10.1128/mSphere.00409-20.3 TABLE S1 Increased expression of miRNAs in Scr siRNA-transfected THP-1 cells stimulated with LPS compared with untreated controls. Download Table S1, DOCX file, 0.02 MB. Copyright © 2020 Akino Mercy et al. 2020 Akino Mercy et al. This content is distributed under the terms of the Creative Commons Attribution 4.0 International license.

To further validate these miRNAs as authentic biomarkers for early diagnosis of leptospirosis, we adopted a murine model of LPS stimulation. Time-dependent changes in miRNAs expression profiles were analyzed in serum of mice injected with LPS. Compared to control animals, ∼7, 2, 6, and 5 miRNAs showed >10-fold upregulation at days 4, 7, 14, and 21, respectively (Table S2 and Fig. 2B). Because we were interested in analyzing the miRNAs that are expressed and circulating in serum during early stages of leptospiral infection, we narrowed our miRNA categories to those that were upregulated day 4 and day 7. Of the miRNAs upregulated at day 4 or day 7, three (miR-21-5p, miR-144-3p, and miR-let-7b-5p) shared similarities with the human miRNome profile of the THP1 cells exposed to leptospiral LPS.

10.1128/mSphere.00409-20.4 TABLE S2 Increased expression of miRNAs in mice injected with LPS compared with controls. Download Table S2, DOCX file, 0.02 MB. Copyright © 2020 Akino Mercy et al. 2020 Akino Mercy et al. This content is distributed under the terms of the Creative Commons Attribution 4.0 International license.

### miRNAs for early diagnosis of leptospirosis.

**FIG 3:**
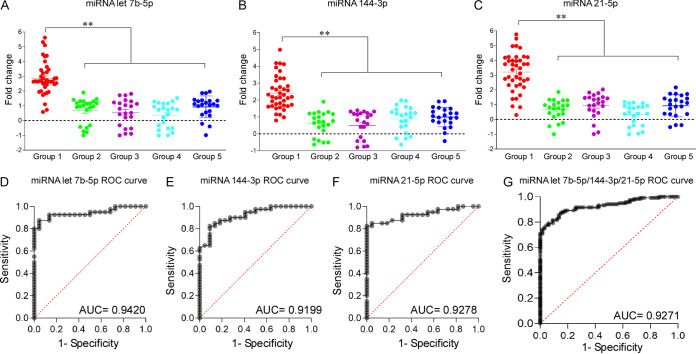
Analysis of the diagnostic potential of identified miRNAs for early diagnosis of leptospirosis. Total RNA was isolated from serum samples of patients and controls. Group 1, patients with laboratory-confirmed cases of leptospirosis; groups 2 to 4, patients suspected of having leptospirosis but identified as having other illnesses, including dengue (group 2), typhoid (group 3), and malaria (group 4); group 5, seronegative healthy controls. (A to C) qPCR analysis was used to measure the fold change in the miRNA levels between confirmed leptospiral cases and controls. The relative fold changes of miR-let-7b-5p (A), miR-144-3p (B), and miR-21-5p (C) showed the diagnostic potential of the identified miRNAs. Data points (dots) represent mean fold changes in individual patient’s serum. The horizontal line represents the cutoff value. **, *P* < 0.005. (D to G) Plots of the sensitivity (true-positive rate) versus 1−specificity (false-positive rate) for miR-let-7b-5p (D), miR-144-3p (E), miR-21-5p (F), and all three miRNAs (G). AUC values indicate the fold change in leptospirosis cases (test group) versus normal samples (control group).

Because miR-21-5p, miR-144-3p, and miR-let-7b-5p shared similarities between human and mouse miRNome profiles and were upregulated during the early stage of leptospiral LPS administration, we next validated these three miRNAs for their diagnostic potential. Semiquantitative PCR was performed with serum samples to validate the diagnostic efficacy of the miRNAs. The fold changes of circulating miR-21-5p, miR-144-3p, and miR-let-7b-5p in the serum of confirmed cases of leptospirosis were significantly higher (*P* < 0.001) than those in healthy controls and persons diagnosed with other febrile illness (Fig. 3A to C).

To confirm the statistical relevance of the miRNA-based serum markers, a receiver operating characteristic (ROC) curve was established, and the area under the curve (AUC) was determined (Fig. 3D to G). AUC values for miR-21-5p, miR-144-3p, miR-let-7b-5p, and all three miRNAs combined were 0.92, 0.91, 0.94, and 0.92 respectively. Quantifying the levels of serum miRNAs is reliable and reproducible, because the sums of the diagnostic sensitivity and specificity were 93.2% and 88.19%, respectively. The high sensitivity and considerable specificity suggest that the signature miRNAs might be potential candidates for diagnosis of acute leptospirosis.

### Functional enrichment analysis of miRNA target genes.

**FIG 4:**
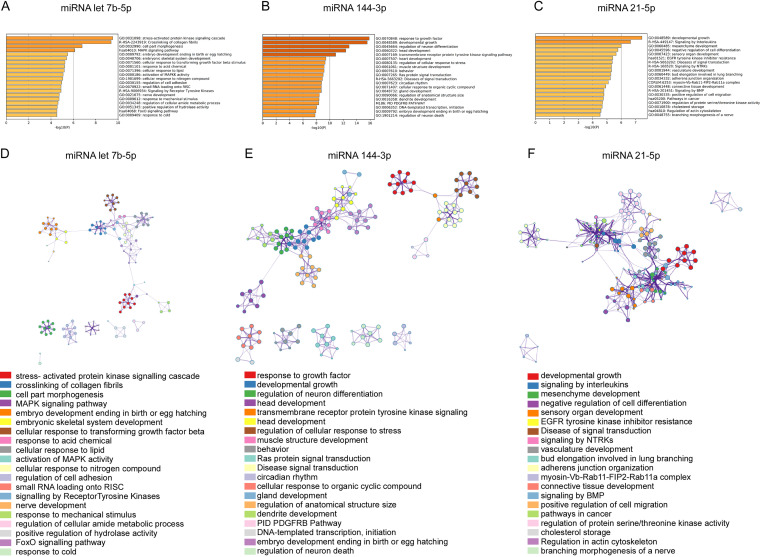
Functional enrichment analysis of miRNA target genes. (A to C) Top 20 clusters from Metascape pathway enrichment analysis of miR-let-7b-5p (A), miR-144-3p (B), and miR-21-5p (C) and the display of the associated genes of miR-let-7b-5p (D), miR-144-3p (E), and miR-21-5p (F) as a network. Nodes of the same color belong to the same cluster, and terms with a similarity score of >0.3 are linked by an edge.

Because these miRNAs have diagnostic potential and were modulated by the TLR2 axis, we next asked whether these miRNAs target the genes that are involved in immune regulation. In both DIANA-microT-CDS and TargetScan, totals of 993, 521, and 1,611 genes were predicted to be the target genes of miR-let-7b-5p, miR-21-5p, and miR-144-3p, respectively. Gene ontology enrichment analysis revealed that miR-21-5p, miR-144-3p, and miR-let-7b-5p play important roles in disease signal transduction, signaling by interleukins, regulation in actin cytoskeleton, stress-activated protein kinase signaling cascade, MAPK signaling pathway, FoxO signaling pathways, cellular response to a transforming growth factor β (TGF-β) stimulus, and small-RNA loading onto the RNA-induced silencing complex (RISC) (Fig. 4A to C). Notably, all these biological processes are interconnected (Fig. 4D to F). Our data imply that the genes involved in the target signaling pathways may serve as potential diagnostic and therapeutic targets for leptospirosis in the future.

## DISCUSSION

Monocytes belong to a subset of circulating white blood cells that can further differentiate into macrophages and dendritic cells (32). *In vivo* and *in vitro* studies have shown that monocytes and their derivatives function as essential components of the innate immune system that mediate host defense and serve as the first line of resistance to microbial attack (33). This innate immunity is triggered by the recognition of pathogen-associated molecular patterns (PAMPs), like LPS, through pattern recognition receptors (PRR); one such conserved PRR is TLR. Indeed, the pleiotropic potential of monocytes suggests that their cellular functions must be tightly regulated in a manner that is distinct from that of more differentiated myeloid cell types to enable appropriate, context-dependent responses.

MicroRNAs (miRNAs) are a large class of small noncoding RNAs that posttranscriptionally regulate mRNAs and subsequently influence essential cellular functions through modulation of gene expression at the RNA or protein level (34). Several miRNAs in myeloid cells were previously identified to impact immune responses, although results have varied due to different cell types, assay methods, and culture conditions (35, 36). Despite differences in cell type, both oligonucleotide-based microarray technology (21, 37, 38) and next-generation sequencing (NGS) technologies (23) indicated that the classic miRNAs hsa-mir-155, hsa-mir-9, and hsa-mir-146a are upregulated in LPS-treated monocytes, suggesting that these miRNAs are critical for promoting pan-myeloid function in response to LPS (21, 23, 37, 38). In another independent study, miRNA profiles of whole blood in mice exposed to LPS demonstrated a significant alteration of the multiple miRNAs (let-7d, miR15b, miR16, miR25, miR92a, miR103, miR107, and miR451) in a dose- and time-dependent fashion. Additionally, LPS from different Gram-negative bacteria, including Escherichia coli, Klebsiella pneumoniae, Pseudomonas aeruginosa, Salmonella enterica, and Serratia marcescens, induced the upregulation of similar miRNAs (24). However, in the present study, stimulation of monocytes (THP1 cells) or whole animals with leptospiral LPS did not induce the upregulation of any of these known signature miRNAs. Rather, 18 different miRNAs (Table S1 and Fig. 2A) were upregulated by stimulation of THP1 cells with leptospiral LPS. Additionally, knocking down TLR2 in THP1 cells normalized the expression of these miRNAs.

The difference in the miRNAs that are upregulated by leptospiral LPS stimulation could be explained by differences in the structure of the leptospiral lipid A. Lipid A is the anchor moiety of LPS in the bacterial membrane and is the active component of LPS responsible for its toxic activity and functions. Although the structure of lipid A from Gram-negative bacteria shows some variability between bacterial species (39), all lipid A moieties are known to stimulate the TLR4 complex. However, leptospiral LPS activated human cells through TLR2 instead of TLR4 (12). The leptospiral lipid A moiety structure was recently deciphered, revealing that it possesses some peculiar characteristics compared to lipid A of Gram-negative bacteria (40), including one phosphate residue that is capped with a methyl group, a lack of a 4′ phosphate group, and four amide-linked acyl chains, longer than in enterobacterial lipid A, with two secondary unsaturated acyl chains. Additionally, of these 18 miRNAs upregulated by leptospiral LPS, 6 (hsa-miR-185-5p, hsa-let-7b-5p, hsa-miR-28-5p, hsa-miR-376c-3p, hsa-miR-195-5p, and hsa-miR-100-5p) were found to be upregulated in bone marrow-derived mesenchymal stem cells stimulated with TLR2 agonist (PAM3CSK4 cells) (41). This further confirms that the identified miRNAs are upregulated by leptospiral LPS through the TLR2 immune axis, and these miRNAs can be significant signature molecules to differentiate leptospiral infection from other bacterial infections with which it is often confused. Because other spirochetes, like *Borrelia* and *Treponema*, do not possess LPS, identification of leptospiral LPS-stimulated miRNAs could differentiate leptospiral infection from other spirochetal infections.

A recent work that examined the microtranscriptome of murine macrophages J774A.1 stimulated with virulent, attenuated, and saprophyte strains of *Leptospira* showed differential expression of 29 miRNAs. Of these 29 miRNAs, the highest fold change was observed for miR155-5p in cells stimulated with pathogenic L. interrogans (42). We did not see an upregulation of this miR155-5p, which could be due to different factors, including but not limited to the difference in the assay method, the cell type, and the culture conditions. Moreover, our study identified the miRNAs that are regulated by LPS stimulation in an *in vivo* mouse model. In this regard, our study is the first to validate the efficacy of miRNAs for the diagnosis of leptospirosis.

Early diagnosis of leptospiral infection is critical for preventing further complications, including multiorgan failure and hemorrhages. Although routine clinical methods, including isolation of leptospires, microscopic agglutination test (MAT), enzyme-linked immunosorbent assay (ELISA), and PCR, are standard methods for diagnosis of leptospirosis, these techniques are time-consuming and require special expertise that might delay treatment. Also, until now, potential biomarkers have included acute phase proteins, cytokines, and chemokines, but they are not sufficient to distinguish other bacterial infections from leptospiral infection. The miRNAs identified in our study are promising biomarkers for early leptospiral infection. In fact, miRNA-based biomarkers are already being used for diagnosis of tuberculosis (43, 44). Because we were interested in developing biomarkers for the early identification of leptospirosis, we characterized the circulating miRNAs in a time-dependent manner (4, 7, 14, and 21 days) in the whole blood of mice after LPS stimulation. The miRNAs miR-21-5p, miR-144-3p, and miR-let 7b-5p shared similarity with the human miRNome profile and were also expressed during the early stage of leptospiral infection (Fig. 2). We validated the diagnostic potential of these three miRNAs using patients’ serum samples. The diagnostic sensitivity and specificity were calculated to be 93.2% and 88.19%, respectively (Fig. 3). Despite accumulating evidence of miRNAs circulating in the blood and body fluids, and the origin and functions of these circulating extracellular miRNAs remain poorly understood (45). Most circulating miRNAs are part of larger lipid or lipoprotein complexes (26, 27, 29, 45). Currently, little is known regarding the biological roles of these molecules at distant sites in the body (46). Extracellular miRNAs may be mediators of cell-cell communications (47, 48) or show hormone-like effects, leading to widespread responses (30, 49). The expression of circulating miRNAs is thought to reflect extrusion of miRNAs from relevant remote tissues or organs or disease processes (30). Indeed, the present study did not show that the miRNAs that are differentially expressed in the circulation are expressed in other tissues. Thus, the source of circulating miRNAs not yet understood, and future studies will be performed to clarify the origin and physiological roles of these circulating miRNAs.

However, attempts were made to identify the physiological role of these miRNAs in cell function using GO analysis. Gene ontology enrichment analysis revealed that miRNAs play important roles in disease signal transduction, signaling by interleukins, the stress-activated protein kinase signaling cascade, the MAPK signaling pathway, FoxO signaling pathways, and cellular response to TGF-β stimulus (Fig. 4A to C). These biological processes are in fact, interconnected (Fig. 4D to F). We propose that these miRNAs fine-tune the gene expression of key components in the pathway under the impact of LPS stimulation. However, this hypothesis does not rule out other possible functions for these miRNAs, since dozens of the targets, validated or predicted, will be still outside the LPS-TLR immune axis pathway. Therefore, in light of the increasing knowledge about the role of miRNAs in disease, the promise they hold for therapeutics and diagnostics should not be underestimated (50). Our study provides evidence that miRNAs play a crucial role in the diagnosis of infectious diseases like leptospirosis that are generally misdiagnosed.

The major challenge of this study is that the miRNAs were tested for its diagnostic potential in a very small patient cohort and without a random sampling scheme. Also, we consider the analysis of miRNAs in the human clinical samples biased, as it is just a confirmation of the serum miRNAs by PCR. In order to use these identified miRNAs as the best prognostic markers, our plan is to enroll a large cohort of patients with laboratory-confirmed leptospirosis. Additionally, comparing the serum miRNA profiles of greater numbers of healthy individuals and patients with other confirmed febrile illnesses will validate the prognostic use of these identified miRNAs. This approach is expected to overcome the problem of definitive diagnosis of acute leptospirosis in the near future.

### Conclusions.

The current work established the relationship between miRNAs and innate immunity and established a detailed profile of circulating miRNAs in THP1 cells and mouse sera after exposure to leptospiral LPS. Further, these findings also established the role of the circulating miRNAs (miR-21-5p, miR-144-3p, and miR-let-7b-5p) as an early diagnostic marker for leptospirosis. These miRNAs will facilitate diagnosis of acute leptospirosis and differentiation of leptospiral infection from other bacterial and spirochetal infection.

## MATERIALS AND METHODS

### Leptospiral strains and media.

The pathogenic leptospiral strains L. interrogans serovar Australis strain AHF651, L. interrogans serovar Autumnalis strain N2, L. kirschneri serovar Grippotyphosa strain D22, L. interrogans serovar Pomona strain H3, and L. borgpetersenii serovar Ballum strain BDU51, isolated from ailing human subjects, and nonpathogenic *L. biflexa* serovar Andamana strain CH11, isolated from water sources, were obtained from the WHO Reference Centre for Leptospirosis, Regional Medical Research Centre, ICMR, Port Blair, Andaman Islands (India). Strains were maintained by regular subculturing in Ellinghausen-McCullough-Johnson-Harris (EMJH) bovine serum albumin-Tween 80 medium (Difco Laboratories, USA) in a shaking incubator (100 rpm) at 30°C. The pathogenicity of the strains was maintained by passaging the isolates in immunocompromised (cyclophosphamide-treated) BALB/c mice ∼15 times (51). These pathogenic mouse-adapted challenge strains (MACS) were used for all the experiments.

### Cells and cell culture.

THP-1 cells obtained from NCCS, Pune, India, were cultured in RPMI medium containing 10% fetal bovine serum (FBS) and 2 mM glutamine (Sigma-Aldrich, St. Louis, MO, USA) and supplemented with 100 U/ml penicillin and 100 μg/ml streptomycin at 37°C in a humidified 5% CO_2_ atmosphere.

### Patients and case definition.

In total, 46 serum samples collected during the early phase of illness (0 to 10 days after the onset of disease) were selected from a bank of samples from 376 laboratory-confirmed cases of leptospirosis (a positive IgM ELISA or MAT titer of ≥1:160 or isolation of leptospires from the blood). A total of 22 seronegative healthy controls matched with respect to age (±5 years) and sex and 66 patients who were hospitalized with a clinical suspicion of leptospirosis and subsequently diagnosed as having other illness, including dengue (22), malaria (22), and typhoid (22), based on laboratory evidence were also included. Informed written consent was obtained from both case patients and controls before sampling, and the study protocol was approved by the Institutional Ethics Committee (IEC) of Bharathidasan University (DM/2014/101/49) as well as receiving permission from the Directorate of Health Services (reference no. 5796/TV 1/07), Tamil Nadu, India.

### Extraction of leptospiral LPS.

Leptospires were collected from mid-log-phase cultures by centrifugation at 10,000 × *g* for 30 min and then washed three times with sterile 1× Dulbecco’s phosphate-buffered saline (PBS) (Corning, Manassas, VA, USA) before LPS extraction. Crude LPS was extracted following standard hot phenol-water extraction as described previously (4). The phenol phase that contained the LPS was dialyzed extensively against Milli-Q water (8 changes for 4 days) to remove phenol. Purified LPS was pelleted by ultracentrifugation (120,000 × *g* for 4 h at 4°C), dissolved in Milli-Q water, and lyophilized. Lyophilized crude material was resuspended in 10 ml of Milli-Q water and treated with DNase and RNase (Sigma-Aldrich, St. Louis, MO, USA), followed by proteinase K digestion (Sigma-Aldrich, St. Louis, MO, USA). LPS was quantified by the phenol-sulfuric acid method using sucrose as a standard (52). Protein was determined by bicinchoninic acid (BCA) kit (Sigma-Aldrich, St. Louis, MO, USA). Total and inorganic phosphorus were determined as described previously. Sodium dodecyl sulfate-polyacrylamide gel electrophoresis (SDS-PAGE) was performed on a 12% polyacrylamide gel using a discontinuous buffer system. The extracted LPS was mixed with 2× SDS-PAGE sample loading buffer (Bio-Rad, Hercules, CA, USA) and boiled for 5 min before loading. Electrophoresis was carried out in a vertical electrophoretic minicell unit (Bio-Rad, Hercules, CA, USA) for 2 h at 100 V using the Tris-glycine running buffer (25 mM Tris, 192 mM glycine, 0.1% SDS [pH 8.3]) (53). A modified silver staining procedure was performed to detect the LPS bands on SDS-PAGE (4, 54) and documented using a gel documentation system (Bio-Rad, Hercules, CA, USA).

### RNA interference and LPS stimulation.

THP-1 cells (0.5 × 10^6^/well) grown in six-well plates were transfected with pools of 4 distinct siRNAs against TLR2 (On-TargetPlus SmartPool; Dharmacon, USA) (50 nM) using the RNAiMAX transfection reagent (Thermo Fisher Scientific, USA). As a control, nontargeting (scrambled) siRNA (Scr siRNA) duplexes (Dharmacon, USA) were used. After 72 h posttransfection, the cells were exposed to 1 μg/ml of leptospiral LPS. Cells and extracellular media were harvested at different time intervals for further experiments.

### Western blotting.

Cell extracts were prepared from THP-1 cells transfected with Scr and TLR2 siRNA using radioimmunoprecipitation assay (RIPA) buffer (50 mM Tris-HCl [pH 7.4], 150 mM NaCl, 0.25% deoxycholic acid, 1 mM EDTA, 1% NP-40, protease and phosphatase inhibitor cocktail; Thermo Scientific). Protein concentrations were quantified using the Pierce 660nm protein assay reagent. Equal amounts of protein (25 μg/well) were separated on 4 to 12% bis-Tris polyacrylamide gels, transferred to a nitrocellulose membrane using a V20 semidry blotter (Scie-Plas, United Kingdom), and probed with antibodies specific for TLR2 (1:1,000; Sigma-Aldrich), TLR4 (1:1,000; Santa Cruz), and GAPDH (1:5,000; Santa Cruz).

### Mice and LPS stimulation.

Ten- to twelve-week-old BALB/c mice were injected intraperitoneally (i.p.) with either PBS or leptospiral LPS (10 μg/g body weight) reconstituted in PBS. After different intervals (0, 4, 7, 14, and 21 days) post-PBS-LPS injection, blood samples with or without heparin were collected by cardiac puncture. Blood samples collected with heparin were gently mixed with RNAlater and used for RNA extraction (cytokine profiling). In order to obtain serum, samples collected without heparin were left overnight at 4°C, and the serum was separated by centrifugation at 3,000 rpm for 5 min and used for total RNA extraction (miRNA profiling). A minimum of 6 mice/cohort were used in the present study. Animal experiments described in this study were carried out in strict accordance with the recommendations approved by the Committee for the Purpose of Control and Supervision on Experiments on Animals (CPCSEA), the Bharathidasan University Ethics Committee in Animal Experimentation (BDU/IAEC/2011/29), and the Bharathidasan University Institutional Biosafety Committee (BT/BS/17/29/2000 PID).

### Cytokine and chemokine profiling.

RNA was isolated from THP1 cells and mouse blood samples using a PureLink RNA minikit or RiboPure blood kit (Thermo Fisher Scientific, Wilmington, DE, USA), respectively. cDNA was synthesized using iScript reverse transcription supermix (Bio-Rad, Hercules, CA, USA). Quantitative reverse transcriptase PCR (qRT-PCR) was performed with a CFX96 Touch real-time PCR detection system (Bio-Rad, Hercules, CA, USA). Semiquantitative real-time PCR was performed using SYBR green master mix (Bio-Rad, Hercules, CA, USA) with a 25-μl reaction volume (50 ng cDNA, 12.5 μl master mix, 0.5 μM concentration of each primer). Primers used in the present study are listed in Table S3. The fold change of each target mRNA was normalized to β-actin and GAPDH.

10.1128/mSphere.00409-20.5 TABLE S3 Primers used in the study. Download Table S3, DOCX file, 0.02 MB. Copyright © 2020 Akino Mercy et al. 2020 Akino Mercy et al. This content is distributed under the terms of the Creative Commons Attribution 4.0 International license.

### Microarrays and miRNA profiling.

Total RNA was isolated from extracellular media of control and TLR2 knockdown cells, THP1 cells, or mouse serum (Norgen Biotek Corp., Canada). Reverse transcription of RNA (500 ng) was performed using a miScript reverse transcription kit (Qiagen, Hilden, Germany). Briefly, with miScript HiSpec buffer, poly(A) tails were added to mature miRNAs and converted to cDNA by reverse transcriptase with oligo(dT) priming. The cDNAs were further used in miRNA profiling using the human and mouse whole miRNome miScript miRNA qPCR arrays (v.16 miRNAs; Qiagen) per the manufacturer’s procedure. All experiments were performed in triplicate.

### Biomarker signature.

For the identification of signature miRNAs expressed during the acute phase of leptospiral infection, we systematically compared miRNA profiles at different days after LPS administration. Day 0, days 4 to 7, and days 14 to 21 were considered control, early, and convalescent phases of leptospiral infection, respectively. To identify miRNAs for early diagnosis of leptospirosis, we devised 5 comparison groups: (i) control versus day 4, (ii) control versus day 7, (iii) control versus day 14, (iv) control versus day 21, and (v) days 4 to 7 versus days 14 to 21.

### Validation of differentially regulated miRNA in clinical cases.

Based on the miRNome miRNA array, we identified three signature miRNAs (miR-21-5p, miR-144-3p, and miR-let-7b-5p) that could have potential for early diagnosis of leptospirosis. We validated the efficacy of these miRNAs using patients’ serum samples. A serum-plasma RNA purification minikit (Norgen Biotek Corp., Canada) was used for the extraction of RNA. The RNA was reverse transcribed using a miScript II RT kit (Qiagen, Hilden, Germany), according to the manufacturer’s protocol. cDNA obtained from the RT reaction was diluted 1:10 with RNase-free water, and real-time qPCR was performed using the miScript SYBR green PCR kit (Qiagen, Hilden, Germany) that contains the miScript universal primer (reverse primer). MystiCq microRNA qPCR assay primers specific for miR-21-5p, miR-144-3p, and miR-let-7b-5p (Sigma-Aldrich, St. Louis, MO, USA) were used as forward primers. miRNA expression was normalized to the internal control SNORD25 (Sigma-Aldrich, St. Louis, MO, USA). Real-time qPCRs were performed at 95°C for 15 min, followed by 40 cycles of 94°C for 15 s, 60°C for 30 s, and 70°C for 30 s. Each reaction was carried out in triplicate in CFX96 detection system (Bio-Rad, Hercules, CA, USA), and data normalization was performed using the ΔΔ*C_T_* method.

### Prediction of target genes.

For miRNAs miR-21-5p, miR-144-3p, and miR-let-7b-5p, we predicted the target genes using two different target prediction databases: TargetScan 7.1 (http://www.targetscan.org/vert_72/) and DIANA-microT-CDS (http://snf-515788.vm.okeanos.grnet.gr/). The threshold was set to 0.7, and we retained only the gene sets that were significant at a nominal *P* value threshold of 0.01.

### Gene ontology enrichment analysis.

Gene ontology (GO) was used for gene functional enrichment analysis using Metascape software (http://metascape.org). The resulting GO terms with *P* values of <0.05 were considered significantly enriched in the differentially expressed miRNAs.

### PPI network.

A PPI network was constructed using Cytoscape software (version 3.6.1) to investigate the role of these miRNAs in the pathogenesis of leptospirosis (55). An integrated score of >0.4 (the default threshold in the STRING database; http://www.string-db.org [56]) was defined to construct the PPI network.

### Statistical analysis.

All data were normally distributed and are presented as means and standard errors of the means (SEM). In the case of single-mean comparisons, data were analyzed by Student's *t* test or, when not normally distributed, a nonparametric Mann-Whitney U test. Differences in means among multiple data sets were analyzed using 1-way analysis of variance (ANOVA) with the Tukey test. *P* values less than 0.05 were considered significant. The diagnostic potential of the miRNAs was evaluated by calculating the area under the receiver operating characteristic (ROC) curve and AUC. For ROC curves, the cutoff value was defined to maximize the sum of sensitivity and specificity. The data were computed with SigmaPlot 11.0 software or GraphPad Prism 8.
