# Supplementary figures and images for: MicroRNAs Regulated by the LPS/TLR2 Immune Axis as Bona Fide Biomarkers for Diagnosis of Acute Leptospirosis
Source: mSphere. 2020 Jul 15;5(4):e00409-20. doi: 10.1128/mSphere.00409-20 (PMC7364213; doi:10.1128/mSphere.00409-20)

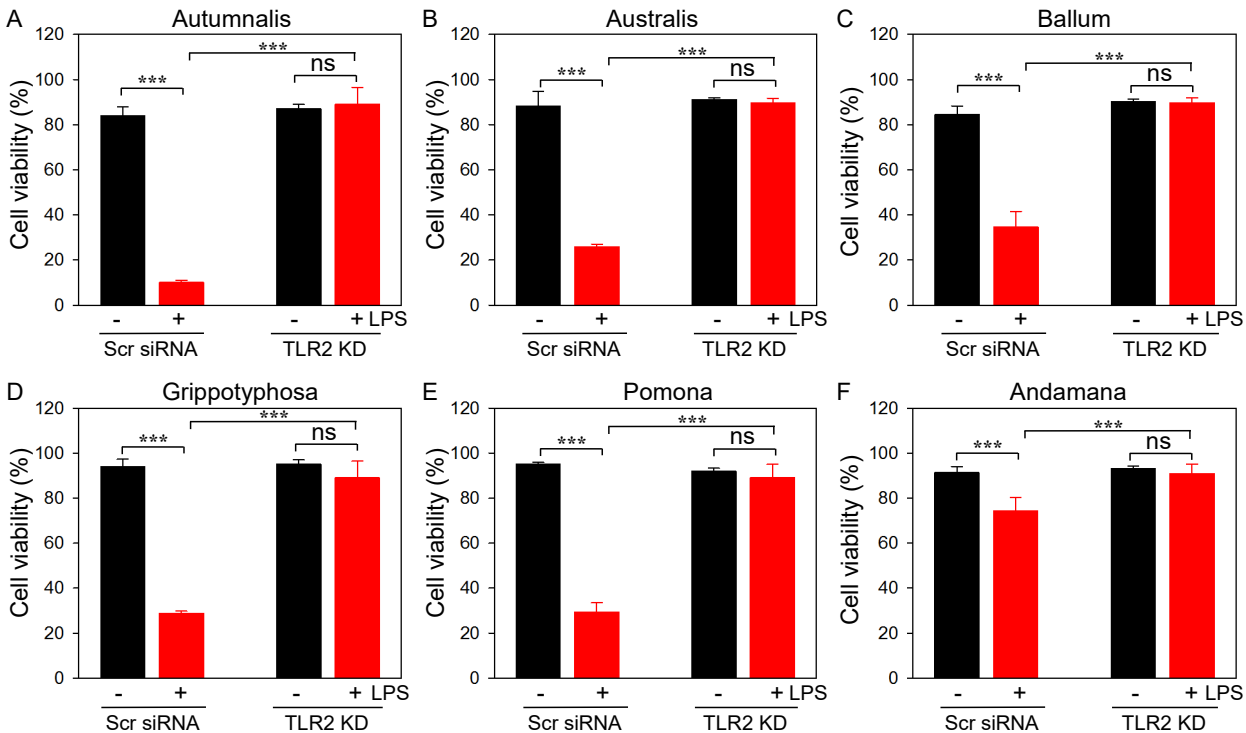

Supplement: FIG S1 [file mSphere.00409-20-sf001.pdf]

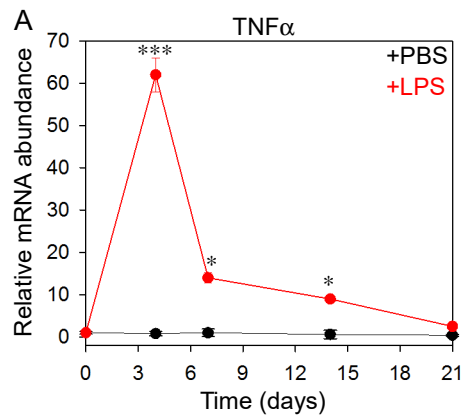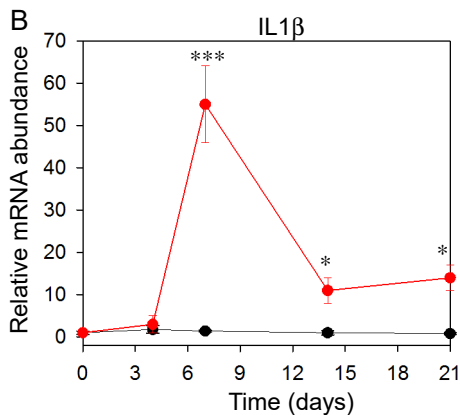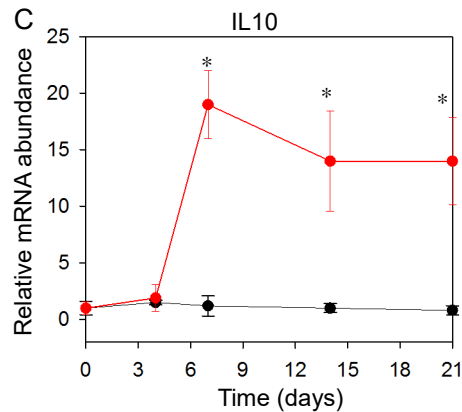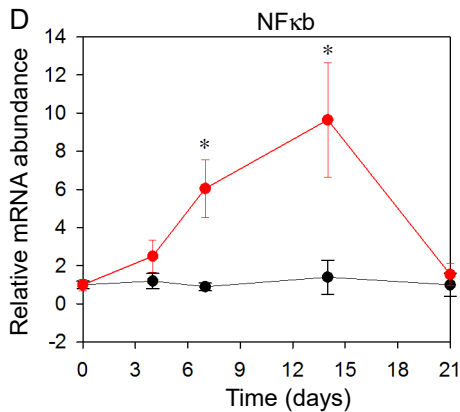

Supplement: FIG S2 [file mSphere.00409-20-sf002.pdf]
