# Supplementary material for: MicroRNAs Regulated by the LPS/TLR2 Immune Axis as Bona Fide Biomarkers for Diagnosis of Acute Leptospirosis
Source: mSphere. 2020 Jul 15;5(4):e00409-20. doi: 10.1128/mSphere.00409-20 (PMC7364213; doi:10.1128/mSphere.00409-20)
Supplement: TABLE S1 [file mSphere.00409-20-st001.docx]

**Supplementary Table 1**

| **No.** | **miRNA Name** | **Fold regulation** |
| --- | --- | --- |
|  | hsa-miR-185-5p | 30.76 |
|  | hsa -miR-425-5p | 11.56 |
|  | hsa -miR-302b-3p | 32.70 |
|  | hsa -miR-21-5p | 14.35 |
|  | hsa -miR-210-3p | 467.53 |
|  | hsa -miR-125b-5p | 93.83 |
|  | hsa -miR-28-5p | 136.01 |
|  | hsa -miR-124-3p | 14.40 |
|  | hsa -miR-376c-3p | 23.81 |
|  | hsa -miR-144-3p | 22.27 |
|  | hsa -miR-151a-5p | 92.29 |
|  | hsa -miR-195-5p | 230.43 |
|  | hsa -miR-302a-3p | 318.66 |
|  | hsa -let-7b-5p | 201.46 |
|  | hsa -miR-128-3p | 12.18 |
|  | hsa -miR-122-5p | 63.43 |
|  | hsa -miR-100-5p | 28.52 |
|  | hsa -miR-302c-3p | 1885.76 |
