# Supplementary material for: MicroRNAs Regulated by the LPS/TLR2 Immune Axis as Bona Fide Biomarkers for Diagnosis of Acute Leptospirosis
Source: mSphere. 2020 Jul 15;5(4):e00409-20. doi: 10.1128/mSphere.00409-20 (PMC7364213; doi:10.1128/mSphere.00409-20)
Supplement: TABLE S2 [file mSphere.00409-20-st002.docx]

**Supplementary Table 2**

| **No.** | **miRNA Name** | **Fold regulation >10** |
| --- | --- | --- |
| **Day 0 Vs Day 4** | | |
|  | mmu-miR-16-5p | 20.51 |
|  | mmu-miR-92a-3p | 28.30 |
|  | mmu-miR-25-3p | 14.67 |
|  | mmu-miR-31-5p | 86.73 |
|  | mmu-miR-30c-5p | 21.07 |
|  | mmu-miR-15b-5p | 15.48 |
|  | mmu-miR-144-3p | 26.86 |
| **Day 0 Vs Day 7** | | |
|  | mmu-miR-16-5p | 19.86 |
|  | mmu-miR-21a-5p | 10.47 |
| **Day 0 Vs Day 14** | | |
|  | mmu-miR-16-5p | 31.28 |
|  | mmu-miR-31-5p | 11.60 |
|  | mmu-miR-10p-5p | 21.78 |
|  | mmu-miR-183-5p | 211.81 |
|  | mmu-miR-1a-3p | 25.72 |
|  | mmu-miR-214-3p | 46.87 |
| **Day 0 Vs Day 28** | | |
|  | mmu-miR-124-3p | 11.21 |
|  | mmu-miR-23b-3p | 15.94 |
|  | mmu-miR-183-5p | 177.74 |
|  | mmu-miR-295-3P | 21.91 |
|  | mmu-miR-214-3P | 50.25 |
| **miRNA that share similarity with THP-1 cells** | | |
| **No.** | **miRNA Name** | **Fold regulation** |
|  | mmu-miR-144-3p | 26.86 |
|  | mmu-miR-21a-5p | 10.47 |
|  | mmu-let-7b-5p | 3.68 |
