# Supplementary material for: MicroRNAs Regulated by the LPS/TLR2 Immune Axis as Bona Fide Biomarkers for Diagnosis of Acute Leptospirosis
Source: mSphere. 2020 Jul 15;5(4):e00409-20. doi: 10.1128/mSphere.00409-20 (PMC7364213; doi:10.1128/mSphere.00409-20)
Supplement: TABLE S3 [file mSphere.00409-20-st003.docx]

**Supplementary Table 3**

| **Gene**  **Name** | **Direction** | **Sequence (5’-3’)** | **Product Size (bp)** |
| --- | --- | --- | --- |
| **For THP-1 mRNAs** | | | |
| TNFα | Forward | CCTCTCTCTAATCAGCCCTCTG | 221 |
|  | Reverse | GAGGACCTGGGAGTAGATGAG |  |
| IL-10 | Forward | GCCCTTTGCTATGGTGTCCT | 189 |
|  | Reverse | TTTTCAGGGATGAAGCGGCT |  |
| NF-κb | Forward | AACAGAGAGGATTTCGTTTCC | 133 |
|  | Reverse | TTTGACCTGAGGGTAAGACTTCT |  |
| IL-1β | Forward | GGACAAGCTGAGGAAGATGC | 249 |
|  | Reverse | TCGTTATCCCATGTGTCGAA |  |
| GAPDH | Forward | AACGACCCCTTCATTGAC | 191 |
|  | Reverse | TCCACGACATACTCAGCAC |  |
| **For murine mRNAs** | | | |
| TNFα | Forward | GGACTAGCCAGGAGGGAGAA | 162 |
|  | Reverse | CGCGGATCATGCTTTCTGTGA |  |
| IL-10 | Forward | GCCCTTTGCTATGGTGTCCT | 179 |
|  | Reverse | TTTTCAGGGATGAAGCGGCT |  |
| NF-κb | Forward | ACCACTGCTCAGGTCCACTGTC | 193 |
|  | Reverse | GCTGTCACTATCCCGGAGTTCA |  |
| IL-1β | Forward | GTATGGGCTGGACTGTTC | 142 |
|  | Reverse | GCTGTCTGCTCATTCACG |  |
| ßActin | Forward | CTGAAGTACCCCATTGAACA | 309 |
|  | Reverse | TCATTGTAGAAGGTGTGGTG |  |
